# Prevalence and prognosis of acute ischemic stroke coexisting with unruptured intracranial aneurysms

**DOI:** 10.3389/fneur.2023.1286193

**Published:** 2023-11-30

**Authors:** Yujia Yan, Xingwei An, Hecheng Ren, Bin Luo, Jin Han, Song Jin, Li Liu, Ying Huang

**Affiliations:** ^1^Academy of Medical Engineering and Translational Medicine, Tianjin University, Tianjin, China; ^2^Department of Neurosurgery, Tianjin Huanhu Hospital, Tianjin, China; ^3^Academy of Clinical Medicine, Inner Mongolia Medical University, Hohhot, China; ^4^Department of Radiology, Tianjin Huanhu Hospital, Tianjin, China

**Keywords:** acute ischemic stroke, unruptured intracranial aneurysms, recurrence, prognosis, risk factors

## Abstract

**Objectives:**

The prevalence of unruptured intracranial aneurysms (UIAs) in the acute ischemic stroke (AIS) cohort is probably higher than in the general population. This study investigated the prevalence of UIAs in AIS patients and the management risk and prognosis when treating AIS.

**Methods:**

From January 2020 to January 2023, we conducted a single-center retrospective study at Tianjin Huanhu Hospital. Each patient underwent both brain MRI and MRA/CTA to diagnose AIS and UIAs. Clinical, radiologic, and therapeutic data during hospitalization and prognosis were analyzed. Propensity-score matching (PSM) was performed to evaluate the risk of in-hospital adverse events, unfavorable outcomes at discharge when receiving post-stroke treatment and stroke recurrence.

**Results:**

In all, 2,181 AIS patients were included, of whom 270 had UIAs (12.4%; 95%CI 11.0–13.8%). From the unmatched and matched cohort, the incidence of in-hospital adverse events and unfavorable outcomes at discharge in patients with UIAs were not significantly different; the risk of stroke recurrence was significantly higher in patients with UIAs than in those without (unmatched: aHR, 1.71 [1.08–2.70]; matched: aHR, 2.55 [1.16–5.58]). Multivariable Cox regression models showed that aneurysm size and the presence of homoregional infarction associated with higher risk of recurrence (unmatched: aHR, 1.31 [1.21–1.41] and aHR, 3.50 [1.52–8.10]; matched: aHR, 1.28 [1.18–1.40]; *p* < 0.001 and aHR, 3.71 [1.12–12.34]).

**Conclusion:**

The UIAs may not increase the risk of in-hospital adverse events and unfavorable outcomes at discharge in receiving post-stroke treatment, but it may associated with a high risk of stroke recurrence.

## Introduction

The prevalence of unruptured intracranial aneurysms (UIAs) is approximately 3.2% in the adult population worldwide (1). This rate is known to vary greatly across different populations, ethnicities and the frequency of angiography scans. In a systematic review and meta-analysis spanning 21 countries, the prevalence of UIAs ranged from 0 to 41.8% (2). In a cross-sectional study from Shanghai, China, the prevalence of UIAs was 7.0% (3). Nonetheless, the detection rate of UIAs appears to be elevated in the acute ischemic stroke (AIS) cohort. According to Korean and the Cornell Acute Stroke Academic Registry (NY) reports, the prevalence of UIAs in AIS patients was 7.7 and 11.4%, respectively, and both were higher than the prevalence in the general population (4, 5).

Data on the prevalence of UIAs in the Chinese population with AIS still needs to be investigated. The overlap of similar underlying cerebrovascular risk factors and increased use of angiography scans may increase the prevalence of incidental aneurysms. Moreover, the prognosis and whether aneurysms increase the incidence of adverse events during hospitalization when treating AIS are still unclear. In the present study, we investigated the prevalence of UIAs among AIS patients and analyzed the epidemiological features and evaluated the management risk and prognosis.

## Methods

### Study design and participants

We retrospectively included 2,432 consecutive adult patients with AIS from the Tianjin Huanhu Hospital Residential Stroke Registry between January 2020 and January 2023. We then screened both the magnetic resonance imaging (MRI) and magnetic resonance angiography (MRA)/computed tomography angiography (CTA) reports of all patients in the registry that mentioned the diagnosis of AIS and UIAs. Clinicians and radiologists rechecked the reports and images to confirm the presence of UIAs. AIS were determined based on the World Health Organization criteria (6). Saccular aneurysms were defined as abnormal focal bulges of cerebral arteries (7). The institutional review board of the hospital’s ethics committee approved the study; the need for written informed consent was waived (No. 2019–34).

**Figure 1:**
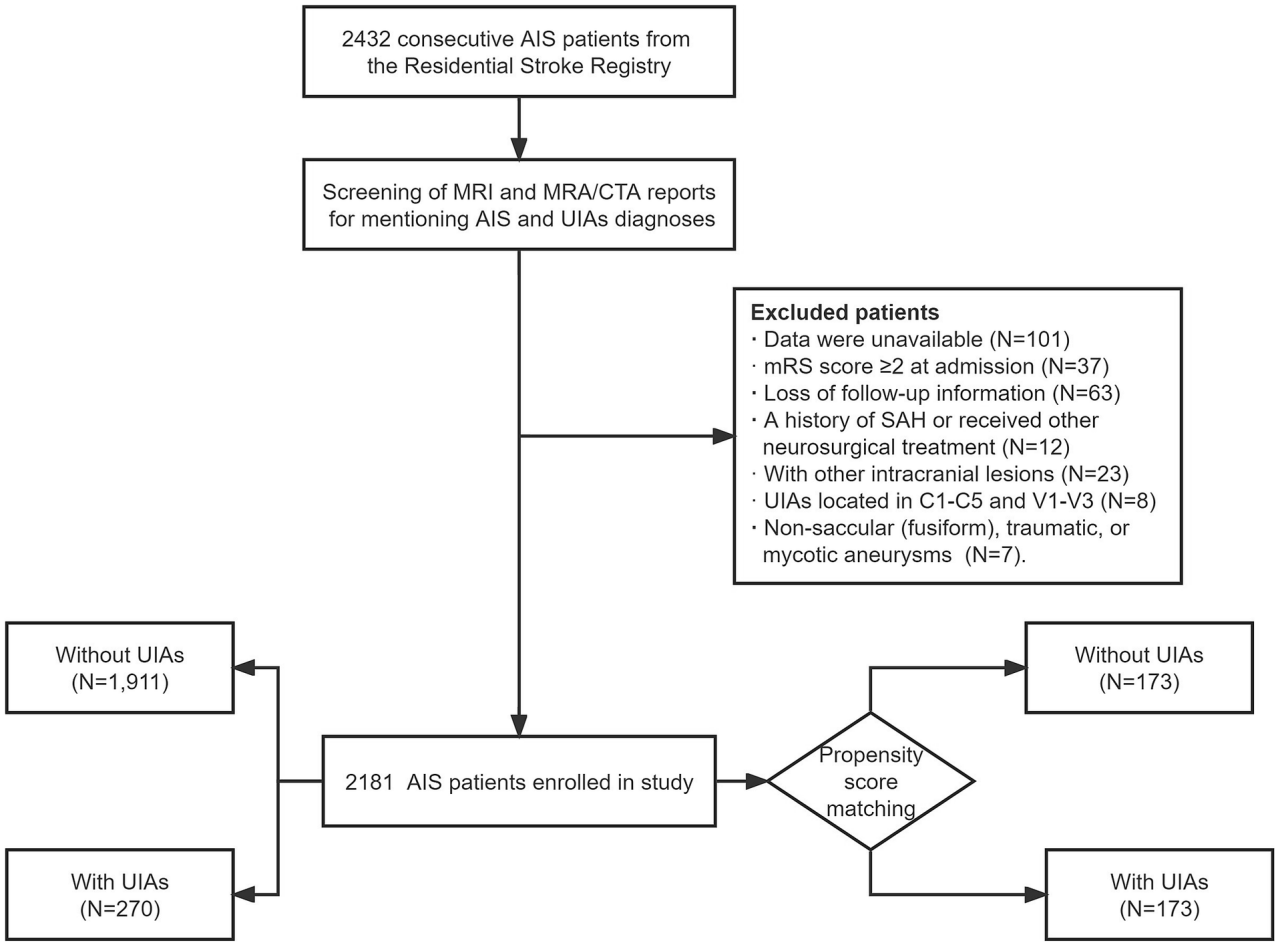
Flowchart of participants’ selection.

Participants were consecutively enrolled. The following exclusion criteria were applied: (1) unavailable or incomplete data; (2) age < 18 years; (3) modified Rankin Scale (mRS) score ≥ 2 at admission; (4) loss of follow-up information; (5) history of subarachnoid hemorrhage (SAH) or receipt of other neurosurgical treatment; (6) presence of other concomitant intracranial lesions such as intracranial tumors or arteriovenous malformation; and (7) UIAs located in C1-C5 and V1-V3 [considered extracranial (8)]. Non-saccular (fusiform), traumatic, or mycotic aneurysms were also excluded considering the difference in etiology and risk of rupture. Overall, 2,181 AIS patients with/without saccular UIAs were included in the study. The flowchart for patient selection is presented in Figure 1.

### Data collection and criteria

Baseline demographics and clinical data including age, sex, and medical history (hypertension, diabetes, hyperlipidemia, coronary artery disease, current smoking, and drinking) were systematically collected. The number (single or multiple), location, sidedness, and size [maximum diameter between any two points on the aneurysm surface, including the neck plane (9)] of aneurysms were recorded.

Etiological subtypes of ischemic stroke were classified according to the Trial of Org 10,172 in Acute Stroke Treatment (TOAST) criteria (10). At the start of the study, we hypothesized that aneurysms are not the cause of stroke. Therapeutic data were obtained through the electronic medical record system. Patients with AIS received standardized treatment including intravenous thrombolysis therapy (IVT), endovascular treatment therapy (EVT)/bridging therapy, anticoagulant therapy, or antiplatelet treatment (11).

**Figure 2:**
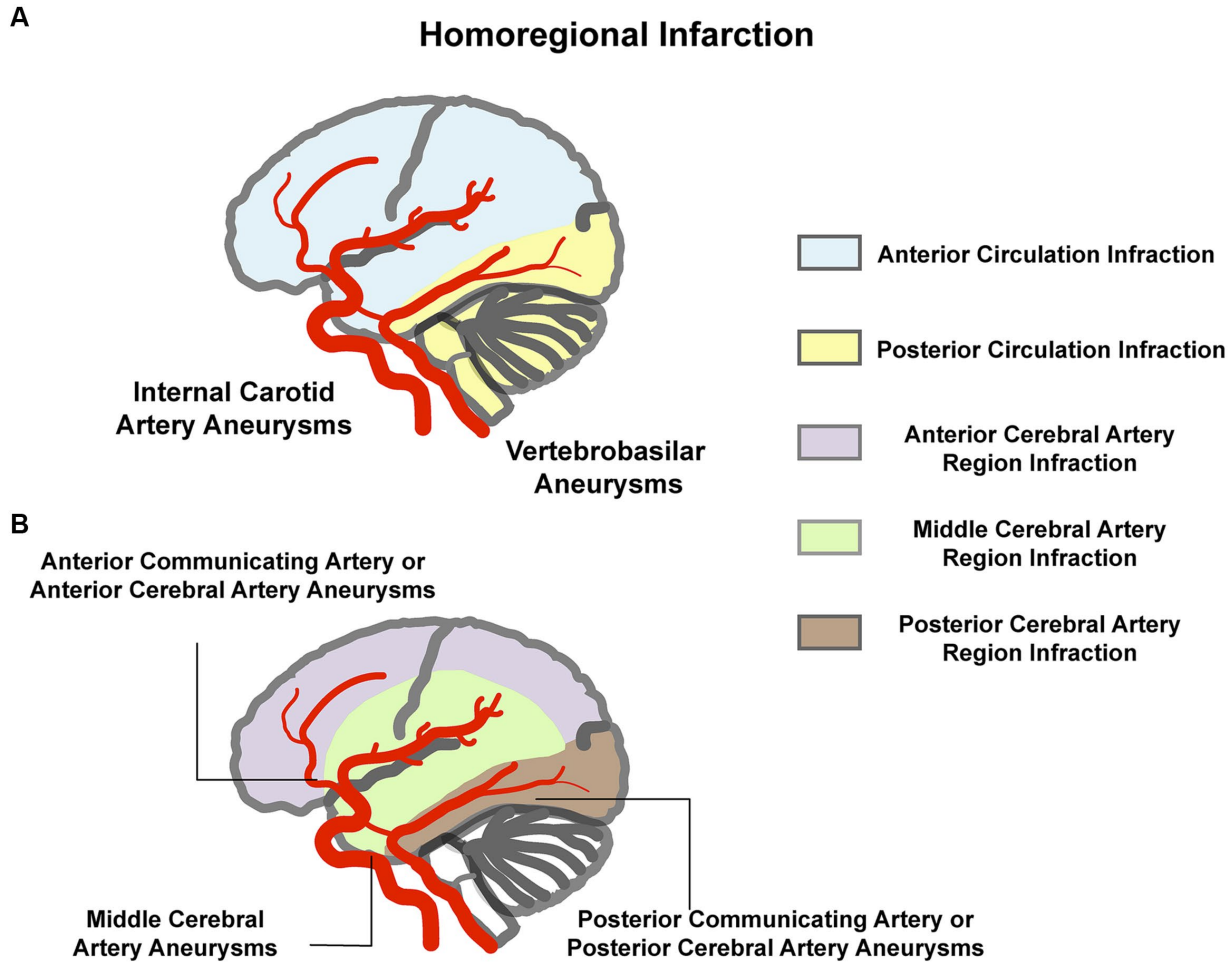
Illustration of “homoregional infarction.” **(A)** Aneurysm in the internal carotid artery or the vertebrobasilar with the ipsilateral anterior circulation or posterior circulation infarction. **(B)** Anterior cerebral artery or anterior communicating artery aneurysm with the ipsilateral or either side anterior cerebral artery region infarction, middle cerebral artery aneurysm with the ipsilateral middle cerebral artery region infarction, and posterior cerebral artery or posterior communicating artery aneurysm with the ipsilateral posterior cerebral artery region infarction.

Based on standard neurovascular anatomical maps, we defined the infarction and aneurysms present with an anatomical homolateral vascular distribution as “Homoregional Infarction.” For example, either side internal carotid artery aneurysm with an ipsilateral cerebral infarction of anterior cerebrovascular circulation, as shown in Figure 2.

### Outcomes

The primary outcomes were unfavorable outcome (mRS score 3–6) at discharge (12) and in-hospital adverse events including in-hospital mortality, infarct enlargement, and intracranial hemorrhages (ICHs), either symptomatic or asymptomatic, detected during post-stroke treatment CT or MRI (13). The secondary outcome was stroke recurrence. Recurrent stroke is defined as a neurological deficit caused by a new infarct or the worsening of a previous neurological deficit and meets the definition of an ischemic stroke (14).

Follow-up information was acquired through telephone, radiographic assessments, and rehospitalization records after 3 months of discharge, and the study endpoint was April 2023. The mean follow-up duration was 17.8 (median 16.7, range 3.0–37.6) months. The distribution of follow-up time is shown in Supplementary Figure S1.

### Statistical analyses

Shapiro–Wilk test was used to assess the normality. Continuous variables are reported as mean with SD or median (interquartile ranges, IQR), and categorical variables are reported as frequencies (percentages). We used independent *t*-test or Mann–Whitney *U* test for continuous variables and Pearson’s chi-squared and Fisher’s exact tests for categorical variables to compare the groups. Kruskal–Wallis test was used to evaluate the difference in aneurysm size among stroke pathologic subtypes. Propensity-score matching (PSM) was performed to address potential confounding effects between the patients with and without UIAs. The following variables were included in the matching: age; sex; vascular risk factors (hypertension, diabetes mellitus, hyperlipidemia, coronary artery disease, current drinking, and smoking); previous stroke; stroke subtype; and post-stroke therapy. We used 1:1 matching without replacement and used the nearest-neighbor algorithm with a caliper width of 0.01 of the propensity score. Standardized mean differences (SMD) were used to assess the balance condition before and after matching. An SMD <0.1 for a given variable indicates a relatively minor degree of imbalance (15). In total, 173 pairs were included in the PSM cohort, and a good balance was achieved in most variables (shown in the matched cohort, Table 1). Kaplan–Meier curves were plotted for the AIS patients with/without UIAs in the matched and unmatched cohort, respectively, as were comparable cumulative incidence curves accounting for the risk of recurrence. The curves for the groups were compared using the log-rank test. Hazard ratios (HR) with 95% CIs were calculated on univariable (unadjusted) Cox regression models. Adjusted HRs (aHR) were calculated on multivariable Cox regression models. Two-sided *p* < 0.05 were considered to indicate statistically significant differences. PSM was carried out using R 4.2.1, and other data were analyzed using SPSS v.25.0 (IBM Corporation, Armonk, NY, United States) statistical software.

**Table 1:** Demographic and clinical characteristics of AIS patients stratified by the presence of UIAs in the matched and unmatched cohort.

	Unmatched cohort				Matched cohort			
Characteristics	Without UIAs (*n* = 1911)	With UIAs (*n* = 270)	*P-*value	SMD	Without UIAs (*n* = 173)	With UIAs (*n* = 173)	*P*-value	SMD
Age, y, mean (SD)	63.2 (10.9)	65.0 (10.5)	0.009*	0.17	64.1 (10.0)	63.5 (11.0)	0.65	0.05
Female sex, n (%)	566 (29.6)	131 (48.5)	<0.001*	0.40	68 (39.3)	64 (37.0)	0.74	0.05
Risk factors, n (%)								
Hypertension	1,211 (63.4)	226 (83.7)	<0.001*	0.47	138 (79.8)	133 (76.9)	0.60	0.07
Diabetes mellitus	729 (38.1)	93 (34.4)	0.240	0.08	72 (41.6)	58 (33.5)	0.15	0.17
Hyperlipidemia	1,105 (57.8)	224 (83.0)	<0.001*	0.57	136 (78.6)	134 (77.5)	0.90	0.03
Coronary artery disease	755 (39.5)	103 (38.1)	0.669	0.03	66 (38.2)	64 (37.0)	0.91	0.02
Current drinking	578 (30.2)	86 (31.9)	0.591	0.04	47 (27.2)	57 (32.9)	0.29	0.13
Current smoking	916 (47.9)	179 (66.3)	<0.001*	0.38	101 (58.4)	102 (59.0)	>0.999	0.01
Previous stroke, n (%)	335 (17.5)	71 (26.3)	0.001*	0.21	38 (22.0)	36 (20.8)	0.90	0.03
Stroke subtype (TOAST), n (%)			<0.001*	0.63			0.33	0.23
LAA	964 (50.4)	92 (34.1)			80 (46.2)	62 (35.8)		
SAO	567 (29.7)	156 (57.8)			76 (43.9)	90 (52.0)		
CE	215 (11.3)	12 (4.4)			11 (6.4)	12 (6.9)		
UND	139 (7.3)	6 (2.2)			5 (2.9)	6 (3.5)		
Others	26 (1.3)	4 (1.5)			1 (0.6)	3 (1.7)		
Post-stroke therapy, n (%)			<0.001*	1.03			0.89	0.05
IVT	724 (37.9)	55 (20.4)			47 (27.2)	49 (28.3)		
EVT/Bridging therapy	556 (29.1)	10 (3.7)			12 (6.9)	10 (5.8)		
Anticoagulant/Antiplatelet treatment	631(33.0)	205 (75.9)			114 (65.9)	114 (65.9)		

Data expressed as mean (SD), or n (%). AIS, acute ischemic stroke; UIAs, unruptured intracranial aneurysms; SMD, standardized mean differences; CE, cardioembolism; LAA, large-artery atherosclerosis; SAO, small-artery occlusion; UND, stroke of undetermined cause; Others, stroke of other determined etiology; TOAST, Trial of ORG 10172 in Acute Stroke Treatment; IVT, intravenous thrombolysis therapy; EVT, endovascular treatment therapy. *p < 0.05 was considered statistically significant.

## Results

### Characteristics of study participants

In total, 2,181 AIS patients were included in the study, and 270 (12.4, 95%CI, 11.0–13.8%) had at least one saccular aneurysm. The demographic and clinical characteristics of patients (unmatched cohort) were shown in Table 1. The prevalence of cerebrovascular risk factors (hypertension, hyperlipidemia, and current smoking) and history of previous stroke was significantly higher among UIAs patients than in the non-UIAs.

**Figure 3:**
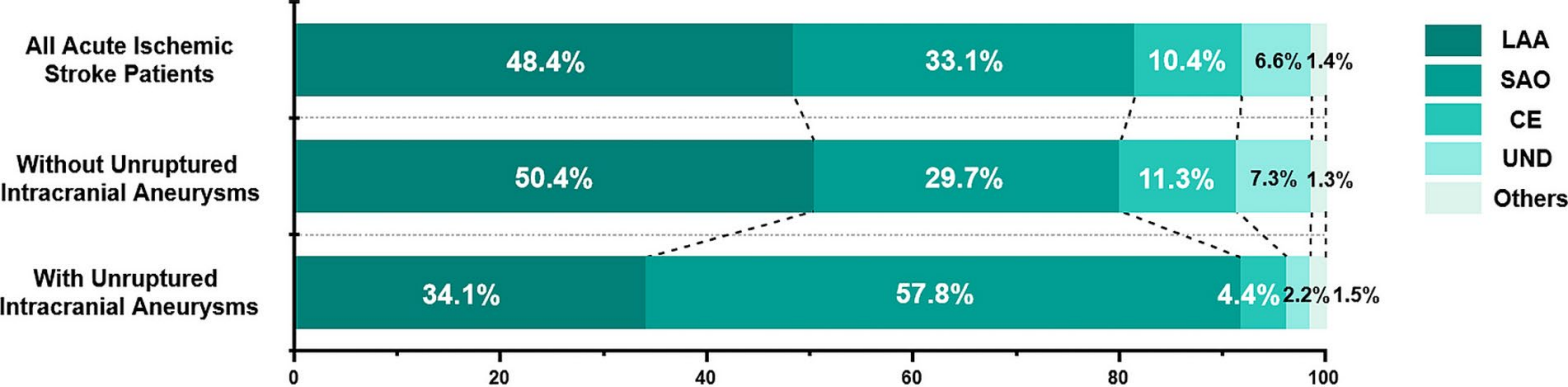
Distribution of etiological subtypes of ischemic stroke based on the TOAST criteria. TOAST, Trial of ORG 10172 in Acute Stroke Treatment; LAA, large-artery atherosclerosis; SAO, small-artery occlusion; CE, cardioembolism; UND, stroke of undetermined cause; Others, stroke of other determined etiology.

Pathological subtypes of all patients were as follows: Large-artery atherosclerosis (LAA), 1,056 (48.4%); small-artery occlusion (SAO), 723 (33.1%); cardioembolism (CE), 227 (10.4%); stroke of undetermined cause (UND), 145 (6.6%); and stroke of other determined etiology (others), 30 (1.4%). The proportion of SAO was significantly higher in patients with UIAs (57.8%, *p* < 0.001, Figure 3) than in those without. Subtypes analysis are shown in Supplementary Table S1. In the SAO subgroup, patients with UIAs had a significantly higher prevalence of risk factors (hypertension, hyperlipidemia, diabetes mellitus, and current smoking) and history of pervious stroke than those without UIAs. The most common location was the internal carotid artery in each subtype, and there was no significant difference in aneurysm size (*p* = 0.298).

### Outcome assessment

**Figure 4:**
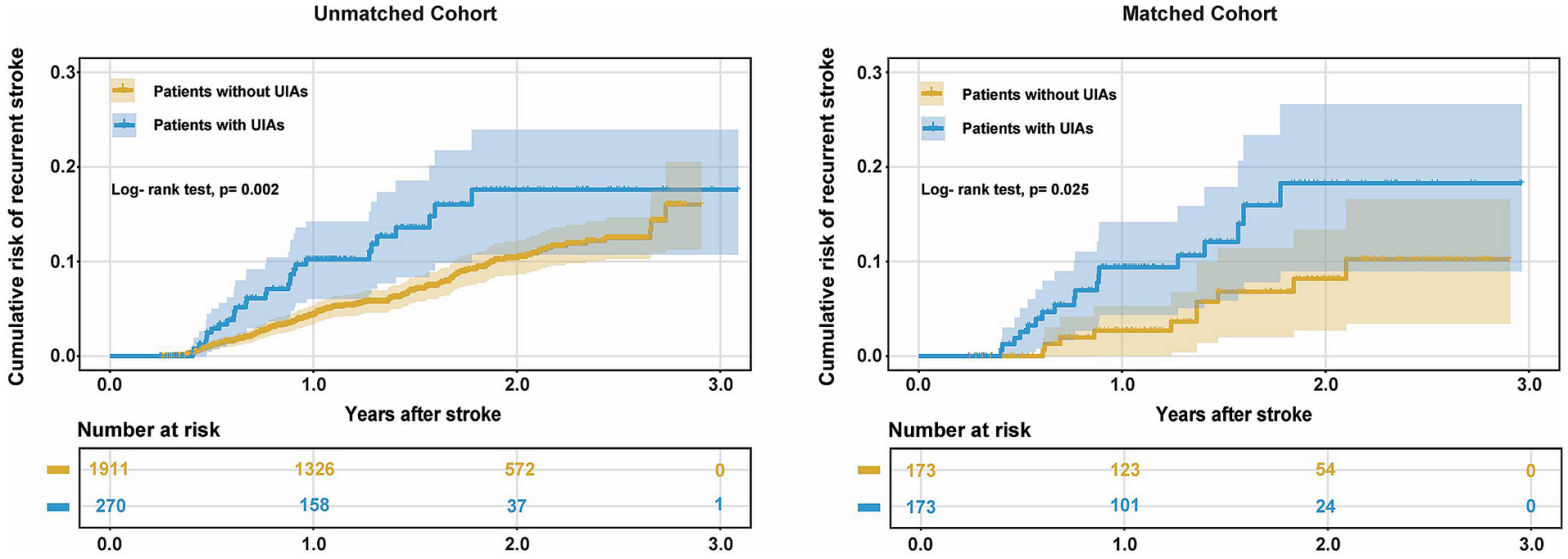
Cumulative risk of recurrence (1-Kaplan–Meier estimates) for AIS patients with UIAs in the unmatched and matched cohort.

The incidence of in-hospital adverse events and unfavorable outcome at discharge in patients with UIAs were not significantly different from those without UIAs in the matched and unmatched cohort (Table 2). Kaplan–Meier estimates of cumulative risk of recurrence are shown in Figure 4. The incidence of stroke recurrence was significantly higher in patients with UIAs than in those without in the unmatched group (32/270 [11.9%] vs. 144/1911 [7.5%]; *p* = 0.002) and matched (21/173 [12.1%] vs. 10/173 [5.8%]; *p* = 0.029) cohorts, respectively. Multivariable Cox regression models also showed a consistent association between UIAs and stroke recurrence in both cohorts (aHR, 1.71 [1.08–2.70]; 2.55 [1.16–5.58]).

**Table 2:** Risk of outcomes in the matched and unmatched cohort stratified by the presence of UIAs.

Outcomes	Without UIAs	With UIAs	*P-*value	Unadjusted odds ratio (95% CI)	Unadjusted hazard ratio (95% CI)	Adjusted hazard ratio (95% CI)^†^
Unmatched cohort, n (%)						
Unfavorable outcome at discharge	142 (7.4)	22 (8.1)	0.676	1.11 (0.69–1.77)		
In-hospital adverse events	111 (5.8)	16 (5.9)	0.939	1.02 (0.60–1.75)		
Stroke recurrence	144 (7.5)	32 (11.9)	0.002*		1.87 (1.25–2.79)	1.71 (1.08–2.70)
Matched cohort, n (%)						
Unfavorable outcome at discharge	9 (5.3)	12 (6.9)	0.501	1.36 (0.56–3.31)		
In-hospital adverse events	5 (2.9)	7 (4.0)	0.560	1.42 (0.44–4.55)		
Stroke recurrence	10 (5.8)	21 (12.1)	0.029*		2.38 (1.09–5.17)	2.55 (1.16–5.58)

Data expressed as n (%). UIAs, unruptured intracranial aneurysms. *p < 0.05 was considered statistically significant. ^†^Adjusting for age, sex, hypertension, hyperlipidemia, current Smoking, previous stroke, TOAST, and post-stroke therapy.

### UIAs and stroke recurrence

Of the 270 patients with UIAs, stroke recurrence was recorded in 32 patients (11.9, 95%CI, 8.0–15.7%). Cox regression models of stroke recurrence was performed as shown in Table 3. Aneurysm size and homoregional infarction were associated with higher risk of recurrence in both cohorts. Adjusting for age, sex, hypertension, hyperlipidemia, current smoking, previous stroke, TOAST, and post-stroke therapy, multivariable Cox regression models also showed a consistent association in the unmatched (aHR, 1.31 [1.21–1.41]; *p* < 0.001 and aHR, 3.50 [1.52–8.10]; *p* = 0.003) and matched (aHR, 1.28 [1.18–1.40]; *p* < 0.001 and aHR, 3.71 [1.12–12.34]; *p* = 0.032) cohorts.

**Table 3:** Cox regression models of stroke recurrence in the matched and unmatched cohort.

	Unmatched cohort		Matched cohort	
Aneurysm characteristic	HR (95% CI)	aHR (95% CI)^†^	HR (95% CI)	aHR (95% CI)^†^
Location				
ICA	Reference		Reference	
ACA/ACoA	1.66 (0.61–4.55)		1.99 (0.61–6.47)	
MCA	0.83 (0.28–2.49)		1.24 (0.38–4.05)	
PCoA	NA		NA	
PC	2.75 (0.99–7.56)		2.58 (0.56–11.94)	
Size	1.23 (1.18–1.29)	1.31 (1.21–1.41)	1.22 (1.16–1.29)	1.28 (1.18–1.40)
Multiple				
No	Reference		Reference	
Yes	0.97 (0.23–4.09)		2.15 (0.49–9.54)	
Homoregional infarction				
No	Reference		Reference	
Yes	3.30 (1.59–6.85)	3.50 (1.52–8.10)	2.97 (1.13–7.82)	3.71 (1.12–12.34)

ICA, internal carotid artery; ACA/ACoA, anterior cerebral artery or anterior communicating artery; MCA, middle cerebral artery; PCoA, posterior communicating artery; PC, posterior circulation. ^†^Adjusting variables include age, sex, hypertension, hyperlipidemia, current Smoking, previous stroke, TOAST, and post-stroke therapy.

## Discussion

To our knowledge, this is the first single-center study in China to report the prevalence of UIAs in AIS patients (12.4%), which is higher than that reported for the general population in existing studies. Our study found that UIAs may not increase the risk of in-hospital adverse events and unfavorable outcome at discharge when receiving post-stroke treatment. However, the incidence of stroke recurrence was significantly higher in patients with UIAs, which was associated with aneurysm size and homoregional infarction.

The prevalence of UIAs varies widely among different populations and ethnicities. Several studies have reported different detection rates of UIAs in AIS patients, ranging from 3.7 to 11.4%, which is equal to or higher than that in the general population (4, 5, 16–19). Although there was a lack of data on the prevalence of UIAs in the Chinese population in the same period, referring to the results of existing studies, the prevalence of UIAs in Chinese people with AIS was higher than the general population (12.4% vs. 7.0%) (3). Similarly, the proportion of stroke subtypes varied according to ethnicity. Large-artery atherosclerosis or small-artery occlusion was more prevalent in Chinese (Asian) cohorts, whereas in European and North American subjects, emboli originating from the heart or extracranial arteries were predominant (20, 21).

The formation of aneurysms is a combination of multiple factors and a progressive process, including hemodynamic stress, vascular risk factors, congenital abnormalities, and genetic predisposition (7, 22). Non-saccular aneurysms have a unique pathogenesis and are different from saccular aneurysms (23), so we did not include them in our analysis. The prevalence of vascular risk factors (including hypertension, hyperlipidemia, and current smoking) in AIS plus UIAs was significantly higher in than in the non-UIAs population (Table 1, unmatched cohort). Hypertension, smoking, and lipid accumulation contribute to the degeneration or disruption of the internal elastic lamina, disrupting the physiological homeostasis of a cerebral artery wall, leading to abnormal blood flow and promoting the formation of aneurysms (22, 24). However, current evidence only indicates a correlation between AIS and UIAs and cannot infer cause and effect. Thus, the phenomenon of a high prevalence of small artery occlusion-related strokes in patients with UIAs, we cannot infer that may be the result of micro thrombosis within the aneurysm (25). Care should be taken regarding the causality and potential mechanisms between AIS and UIAs.

The coexistence of ischemic and hemorrhagic diseases presents more complex and contradictory clinical problems, which may result in a similar effect of 1 + 1 > 2. Did post-stroke treatment increase the risk of ICHs in UIAs? Did untreated aneurysms increase the risk of ischemic stroke recurrence? The answers to such questions are unclear. It is predictable that the detection rate of UIAs will likely continue to rise; hence, appropriate methods to deal with the coexistence of UIAs and AIS needs urgent addressal.

The primary findings of this study were: UIAs may not increase the risk of in-hospital adverse events and unfavorable outcomes at discharge when receiving post-stroke treatment. Aneurysm wall inflammation is thought to play a pivotal role in aneurysm rupture (26). Histological evaluation showed that the aneurysm wall infiltration was prone to rupture (22). Following cerebral artery occlusion, ischemic injury induces a multi-pathway molecular cascade of responses, the inflammatory responses are induced by reactive oxygen species (ROS) after the process of ischemia–reperfusion (27, 28). Therefore, in theory, the risk of bleeding appears to be higher in AIS patients with aneurysms. It is worth noting that classically, UIAs and other untreated intracranial vascular malformations have been considered absolute exclusion criteria. However, in the updated AHA/ASA guidelines for patients known to harbor UIAs <10 mm, IVT is reasonable and probably recommended (11). The results of in-hospital adverse events showed no difference between patients with and without UIAs, and aneurysms did not increase the unfavorable outcome at discharge. This seems to support the guideline recommendations. It may be related to the ability of aspirin reducing the inflammatory response of the arterial wall and may have a protective effect on the aneurysm rupture (29). However, it is notable that in this study, 95.5% of the aneurysms were < 10 mm. Thus, it is reasonable to conclude that aneurysms <10 mm may not increase the risk of adverse events during hospitalization and the unfavorable outcome for post-stroke treatment. The safety of post-stroke treatment with aneurysms larger than 10 mm is unclear.

The second question about stroke recurrence. In our research, the incidence of stroke recurrence was significantly higher in patients with UIAs. Neurosurgeons have been aware of the phenomenon of suspected aneurysmal stroke for two decades and have described this ischemic event as “symptomatic cerebral aneurysms.” However, there was no more explanation of the ischemic event (30). After that, the reports of “Suspected Aneurysmal Stroke” were mainly in the form of case reports (31, 32). The results of this study have shown that stroke recurrence is positively correlated with aneurysm size and the presence of homoregional infarction (Table 3). However, we have no direct evidence of the origin of the emboli, and we cannot exclude whether the source of the emboli was an aneurysm. The optimal management of AIS patients with UIAs is uncertain, and we would not recommend surgical treatment just to avoid stroke recurrence. We preferred to emphasize the necessity of secondary prevention, targeting vascular risk factors, especially hypertension and hyperlipidemia, which occur significantly in the Chinese population. Sodium restriction, balanced diet, and smoking cessation are recommended. Monitoring of unruptured aneurysms is necessary (by CTA or MRA), and aneurysms with enlargement during follow-up should be considered for treatment in the absence of serious complications.

Our study has some limitations. First, we did not provide direct evidence to confirm the source of the emboli. Therefore, this study cautious to conclude the relevant inferences rather than cause-and-effect conclusions. Second, the size of aneurysms was predominantly <10 mm, which lacks representativeness, and the conclusions from this study should be interpreted with caution. Third, with the inherent defects of retrospective analysis. The etiological subtypes of ischemic stroke were obtained from the electronic medical records and secondary assessment by physicians. It was limited by incomplete data, as some patients did not undergo echocardiography and the proportion of cardioembolism may be underestimated. We need to perform a larger, multi-center prospective study to validate the results.

## Conclusion

The overlap of vascular risk factors underlying UIA and AIS makes the prevalence of UIAs seem higher in AIS patients than in the general population. UIAs may not increase the risk of in-hospital adverse events and unfavorable outcomes at discharge when receiving post-stroke treatment. However, the incidence of stroke recurrence was significantly higher in patients with UIAs. We recommend more strict vascular risk factor control and follow-up management of AIS patients with aneurysms.

## Data availability statement

The raw data supporting the conclusions of this article will be made available by the authors, without undue reservation.

## Ethics statement

The Tianjin Huanhu Hospital Ethics Committee [No. 2019(34)] approved the data collection procedures that involved the study participants to ensure that they are conducted in accordance with the ethical standards. Written informed consent for participation was not required for this study in accordance with the national legislation and the institutional requirements.

## Author contributions

YY: Conceptualization, Data curation, Investigation, Methodology, Software, Writing – original draft, Writing – review & editing. XA: Conceptualization, Data curation, Investigation, Methodology, Software, Writing – original draft, Writing – review & editing. HR: Data curation, Methodology, Project administration, Resources, Writing – review & editing. BL: Data curation, Methodology, Writing – review & editing. JH: Formal analysis, Methodology, Software, Writing – review & editing. SJ: Data curation, Methodology, Project administration, Supervision, Writing – review & editing. LL: Data curation, Methodology, Project administration, Supervision, Writing – review & editing. YH: Funding acquisition, Project administration, Resources, Supervision, Validation, Visualization, Writing – review & editing.

## Supplementary material

SUPPLEMENTARY TABLE S1 Differences between stroke pathological subtypes stratified by the presence of UIAs.

SUPPLEMENTARY FIGURE S1 Kernel density estimation-based distribution of follow-up times.

## References

[1] Thompson BG Brown RD Jr Amin-Hanjani S Broderick JP Cockroft KM Connolly ES Jr. Guidelines for the management of patients with unruptured intracranial aneurysms: a guideline for healthcare professionals from the American Heart Association/American Stroke Association. Stroke. (2015) 46:2368–400. doi: 10.1161/str.0000000000000070, PMID: 26089327

[2] Vlak MH Algra A Brandenburg R Rinkel GJ. Prevalence of unruptured intracranial aneurysms, with emphasis on sex, age, comorbidity, country, and time period: a systematic review and meta-analysis. Lancet Neurol. (2011) 10:626–36. doi: 10.1016/s1474-4422(11)70109-0, PMID: 21641282

[3] Li MH Chen SW Li YD Chen YC Cheng YS Hu DJ. Prevalence of unruptured cerebral aneurysms in Chinese adults aged 35 to 75 years: a cross-sectional study. Ann Intern Med. (2013) 159:514–21. doi: 10.7326/0003-4819-159-8-201310150-00004, PMID: 24126645

[4] Kim JH Suh SH Chung J Oh YJ Ahn SJ Lee KY. Prevalence and characteristics of unruptured cerebral aneurysms in ischemic stroke patients. J Stroke. (2016) 18:321–7. doi: 10.5853/jos.2016.00164, PMID: 27488981 PMC5066432

[5] Chen ML Gupta A Chatterjee A Khazanova D Dou E Patel H. Association between unruptured intracranial aneurysms and downstream stroke. Stroke. (2018) 49:2029–33. doi: 10.1161/strokeaha.118.021985, PMID: 30354970 PMC6205209

[6] Adams HP Castel JP Dalai PM Easton JD Gunton RW Hachinski VC.Recommendations on stroke prevention, diagnosis, and therapy. Report of the WHO task force on stroke and other cerebrovascular disorders. Stroke. (1989) 20:1407–31. doi: 10.1161/01.str.20.10.1407, PMID: 2799873

[7] Hurford R Taveira I Kuker W Rothwell PM. Prevalence, predictors and prognosis of incidental intracranial aneurysms in patients with suspected TIA and minor stroke: a population-based study and systematic review. J Neurol Neurosurg Psychiatry. (2021) 92:542–8. doi: 10.1136/jnnp-2020-324418, PMID: 33148817 PMC8053340

[8] Yin Z Zhang Q Zhao Y Lu J Ge P Xie H. Prevalence and procedural risk of intracranial atherosclerotic stenosis coexisting with unruptured intracranial aneurysm. Stroke. (2023) 54:1484–93. doi: 10.1161/strokeaha.122.041553, PMID: 37139814

[9] Skodvin T Johnsen LH Gjertsen Ø Isaksen JG Sorteberg A. Cerebral aneurysm morphology before and after rupture: nationwide case series of 29 aneurysms. Stroke. (2017) 48:880–6. doi: 10.1161/strokeaha.116.015288, PMID: 28265012

[10] Adams HP Jr Bendixen BH Kappelle LJ Biller J Love BB Gordon DL. Classification of subtype of acute ischemic stroke. Definitions for use in a multicenter clinical trial. TOAST. Trial of org 10172 in acute stroke treatment. Stroke. (1993) 24:35–41. doi: 10.1161/01.str.24.1.35, PMID: 7678184

[11] Warner JJ Harrington RA Sacco RL Elkind MSV. Guidelines for the early management of patients with acute ischemic stroke: 2019 update to the 2018 guidelines for the early management of acute ischemic stroke. Stroke. (2019) 50:3331–2. doi: 10.1161/strokeaha.119.027708, PMID: 31662117

[12] Virta JJ Strbian D Putaala J Kaprio J Korja M. Characteristics and outcomes of thrombolysis-treated stroke patients with and without saccular intracranial aneurysms. Stroke. (2022) 53:3616–21. doi: 10.1161/strokeaha.122.040151, PMID: 36254706 PMC9698101

[13] Rücker V Heuschmann PU O'Flaherty M Weingärtner M Hess M Sedlak C. Twenty-year time trends in long-term case-fatality and recurrence rates after ischemic stroke stratified by etiology. Stroke. (2020) 51:2778–85. doi: 10.1161/strokeaha.120.029972, PMID: 32811383

[14] Wang Y Xu J Zhao X Wang D Wang C Liu L. Association of hypertension with stroke recurrence depends on ischemic stroke subtype. Stroke. (2013) 44:1232–7. doi: 10.1161/strokeaha.111.000302, PMID: 23444308

[15] Normand ST Landrum MB Guadagnoli E Ayanian JZ Ryan TJ Cleary PD. Validating recommendations for coronary angiography following acute myocardial infarction in the elderly: a matched analysis using propensity scores. J Clin Epidemiol. (2001) 54:387–98. doi: 10.1016/s0895-4356(00)00321-8, PMID: 11297888

[16] Ishikawa Y Hirayama T Nakamura Y Ikeda K. Incidental cerebral aneurysms in acute stroke patients: comparison of asymptomatic healthy controls. J Neurol Sci. (2010) 298:42–5. doi: 10.1016/j.jns.2010.08.069, PMID: 20864121

[17] Jiranukool J Thiarawat P Galassi W. Prevalence of intracranial aneurysms among acute ischemic stroke patients. Surg Neurol Int. (2020) 11:341. doi: 10.25259/sni_506_2020, PMID: 33194275 PMC7656034

[18] Oh YS Lee SJ Shon YM Yang DW Kim BS Cho AH. Incidental unruptured intracranial aneurysms in patients with acute ischemic. Cerebrovasc Dis. (2008) 26:650–3. doi: 10.1159/000166842, PMID: 18984951

[19] Shono Y Sugimori H Matsuo R Fukushima Y Wakisaka Y Kuroda J. Safety of antithrombotic therapy for patients with acute ischemic stroke harboring unruptured intracranial aneurysm. Int J Stroke. (2018) 13:734–42. doi: 10.1177/1747493018765263, PMID: 29543141

[20] Kim BJ Kim JS. Ischemic stroke subtype classification: an Asian viewpoint. J Stroke. (2014) 16:8–17. doi: 10.5853/jos.2014.16.1.8, PMID: 24741560 PMC3961817

[21] Jacob MA Ekker MS Allach Y Cai M Aarnio K Arauz A. Global differences in risk factors, etiology, and outcome of ischemic stroke in young adults-a worldwide meta-analysis: the GOAL initiative. Neurology. (2022) 98:e573–88. doi: 10.1212/wnl.0000000000013195, PMID: 34906974 PMC8829964

[22] Etminan N Rinkel GJ. Unruptured intracranial aneurysms: development, rupture and preventive management. Nat Rev Neurol. (2016) 12:699–713. doi: 10.1038/nrneurol.2016.150, PMID: 27808265

[23] Krings T Mandell DM Kiehl TR Geibprasert S Tymianski M Alvarez H. Intracranial aneurysms: from vessel wall pathology to therapeutic approach. Nat Rev Neurol. (2011) 7:547–59. doi: 10.1038/nrneurol.2011.136, PMID: 21931350

[24] Frösen J. Smooth muscle cells and the formation, degeneration, and rupture of saccular intracranial aneurysm wall--a review of current pathophysiological knowledge. Transl Stroke Res. (2014) 5:347–56. doi: 10.1007/s12975-014-0340-3, PMID: 24683005

[25] Cannistraro RJ Badi M Eidelman BH Dickson DW Middlebrooks EH Meschia JF. CNS small vessel disease a clinical review. Neurology. (2019) 92:1146–56. doi: 10.1212/wnl.0000000000007654, PMID: 31142635 PMC6598791

[26] Chalouhi N Hoh BL Hasan D. Review of cerebral aneurysm formation, growth, and rupture. Stroke. (2013) 44:3613–22. doi: 10.1161/strokeaha.113.002390, PMID: 24130141

[27] Moskowitz MA Lo EH Iadecola C. The science of stroke: mechanisms in search of treatments. Neuron. (2010) 67:181–98. doi: 10.1016/j.neuron.2010.07.002, PMID: 20670828 PMC2957363

[28] Won SJ Kim JE Cittolin-Santos GF Swanson RA. Assessment at the single-cell level identifies neuronal glutathione depletion as both a cause and effect of ischemia-reperfusion oxidative stress. J Neurosci. (2015) 35:7143–52. doi: 10.1523/jneurosci.4826-14.2015, PMID: 25948264 PMC4420782

[29] Hasan DM Mahaney KB Brown RD Jr Meissner I Piepgras DG Huston J. Aspirin as a promising agent for decreasing incidence of cerebral aneurysm rupture. Stroke. (2011) 42:3156–62. doi: 10.1161/strokeaha.111.619411, PMID: 21980208 PMC3432499

[30] Friedman JA Piepgras DG Pichelmann MA Hansen KK Brown RD Jr Wiebers DO. Small cerebral aneurysms presenting with symptoms other than rupture. Neurology. (2001) 57:1212–6. doi: 10.1212/wnl.57.7.1212, PMID: 11591837

[31] Mokin M Darkhabani Z Binning MJ Levy EI Siddiqui AH. Small unruptured partially thrombosed aneurysms and stroke: report of three cases and review of the literature. J Neurointerv Surg. (2012) 4:e6. doi: 10.1136/neurintsurg-2011-010026, PMID: 21990496

[32] Bhat V Kodapala S. Transient ischemic attack due to unruptured basilar artery aneurysm. Cureus. (2022) 14:e24102. doi: 10.7759/cureus.24102, PMID: 35573510 PMC9103616

