# Supplementary material for: Prevalence and prognosis of acute ischemic stroke coexisting with unruptured intracranial aneurysms
Source: Front Neurol. 2023 Nov 30;14:1286193. doi: 10.3389/fneur.2023.1286193 (PMC10731460; doi:10.3389/fneur.2023.1286193)
Supplement: SUPPLEMENTARY TABLE S1 — Differences between stroke pathological subtypes stratified by the presence of UIAs. [file Table_1.docx]

**Supplemental Table 1** Differences between stroke pathological subtypes stratified by the presence of UIAs.

| **Characteristics** | **LAA** | | | **SAO** | | | **CE** | | | **UND & Others** | | |
| --- | --- | --- | --- | --- | --- | --- | --- | --- | --- | --- | --- | --- |
|  | **Without UIAs**  **(n=964, 50.4%)** | **With UIAs**  **(n=92, 34.1%)** | ***P* Value** | **Without UIAs**  **(n=567, 29.7%)** | **With UIAs**  **(n=156, 57.8%)** | ***P* Value** | **Without UIAs**  **(n=215, 11.3%)** | **With UIAs**  **(n=12, 4.4%)** | ***P* Value** | **Without UIAs**  **(n=165, 8.6%)** | **With UIAs**  **(n=10, 3.7%)** | ***P* Value** |
| Age, y, mean (SD) | 62.9 (10.6) | 65.0 (9.7) | 0.064 | 61.5 (10.5) | 64.4 (10.7) | 0.002* | 64.9 (11.3) | 74.3 (7.7) | 0.005* | 68.6 (11.4) | 63.0 (12.9) | 0.137 |
| Female sex, n (%) | 251 (26.0) | 38 (41.3) | 0.002* | 175 (30.9) | 82 (52.6) | <0.001* | 78 (36.3) | 8 (66.7) | 0.062 | 62 (37.6) | 3 (30.0) | 0.746 |
| Risk factors, n (%) |  |  |  |  |  |  |  |  |  |  |  |  |
| Hypertension | 615 (63.8) | 82 (89.1) | <0.001* | 374 (66.0) | 128 (82.1) | <0.001* | 123 (57.2) | 9 (75.0) | 0.224 | 99 (60.0) | 7 (70.0) | 0.742 |
| Diabetes mellitus | 351 (36.4) | 37 (40.2) | 0.469 | 251 (44.3) | 50 (32.1) | 0.008* | 58 (27.0) | 3 (25.0) | >0.999 | 69 (41.8) | 3 (30.0) | 0.528 |
| Hyperlipidemia | 561 (58.2) | 75 (81.5) | <0.001* | 331 (58.4) | 131 (84.0) | <0.001* | 120 (55.8) | 10 (83.3) | 0.061 | 93 (56.4) | 8 (80.0) | 0.194 |
| Coronary artery disease | 299 (31.0) | 30 (32.6) | 0.753 | 178 (31.4) | 59 (37.8) | 0.130 | 144 (67.0) | 9 (75.0) | 0.564 | 134 (81.2) | 5 (50.0) | 0.032* |
| Current Drinking | 295 (30.6) | 30 (32.6) | 0.690 | 164 (28.9) | 53 (34.0) | 0.223 | 73 (34.0) | 2 (16.7) | 0.345 | 46 (27.9) | 1 (10.0) | 0.292 |
| Current Smoking | 483 (50.1) | 63 (68.5) | 0.001* | 261 (46.0) | 104 (66.7) | <0.001* | 107 (49.8) | 4 (33.3) | 0.268 | 65 (39.4) | 8 (80.0) | 0.018* |
| Previous stroke, n (%) | 184 (19.1) | 22 (23.9) | 0.264 | 66 (11.6) | 42 (26.9) | <0.001* | 42 (19.5) | 3 (25.0) | 0.709 | 43 (26.1) | 4 (40.0) | 0.461 |
| **Number of aneurysms†** |  | 95 |  |  | 159 |  |  | 13 |  |  | 10 |  |
| Location, n (%) |  |  |  |  |  |  |  |  |  |  |  |  |
| ICA |  | 53 (55.8) |  |  | 93 (58.5) |  |  | 9 (69.2) |  |  | 6 (60.0) |  |
| ACA/ACoA |  | 13 (13.7) |  |  | 21 (13.2) |  |  | 1 (7.7) |  |  | 1 (10.0) |  |
| MCA |  | 14 (14.7) |  |  | 29 (18.2) |  |  | 3 (23.1) |  |  | - |  |
| PCoA |  | 2 (2.1) |  |  | 5 (3.1) |  |  | - |  |  | 1 (10.0) |  |
| PC |  | 13 (13.7) |  |  | 11 (6.9) |  |  | - |  |  | 2 (20.0) |  |
| Size, mm, median (IQR) |  | 4.0 (2.2) |  |  | 5.1 (2.3) |  |  | 4.8 (1.8) |  |  | 4.4 (1.5) |  |

Data expressed as mean (SD), n (%), or median (IQR).

Abbreviations: LAA indicates large-artery atherosclerosis; SAO, small-artery occlusion; CE, cardioembolism; UND, stroke of undetermined cause; Others, stroke of other determined etiology; ICA, internal carotid artery; ACA/ACoA, anterior cerebral artery or anterior communicating artery; MCA, middle cerebral artery; PCoA, posterior communicating artery; PC, posterior circulation.

† The number of aneurysms exceeds the patients because of the presence of multiple aneurysms.

**P* < 0.05 was considered statistically significant.
