# Supplementary figures and images for: Prevalence and prognosis of acute ischemic stroke coexisting with unruptured intracranial aneurysms
Source: Front Neurol. 2023 Nov 30;14:1286193. doi: 10.3389/fneur.2023.1286193 (PMC10731460; doi:10.3389/fneur.2023.1286193)

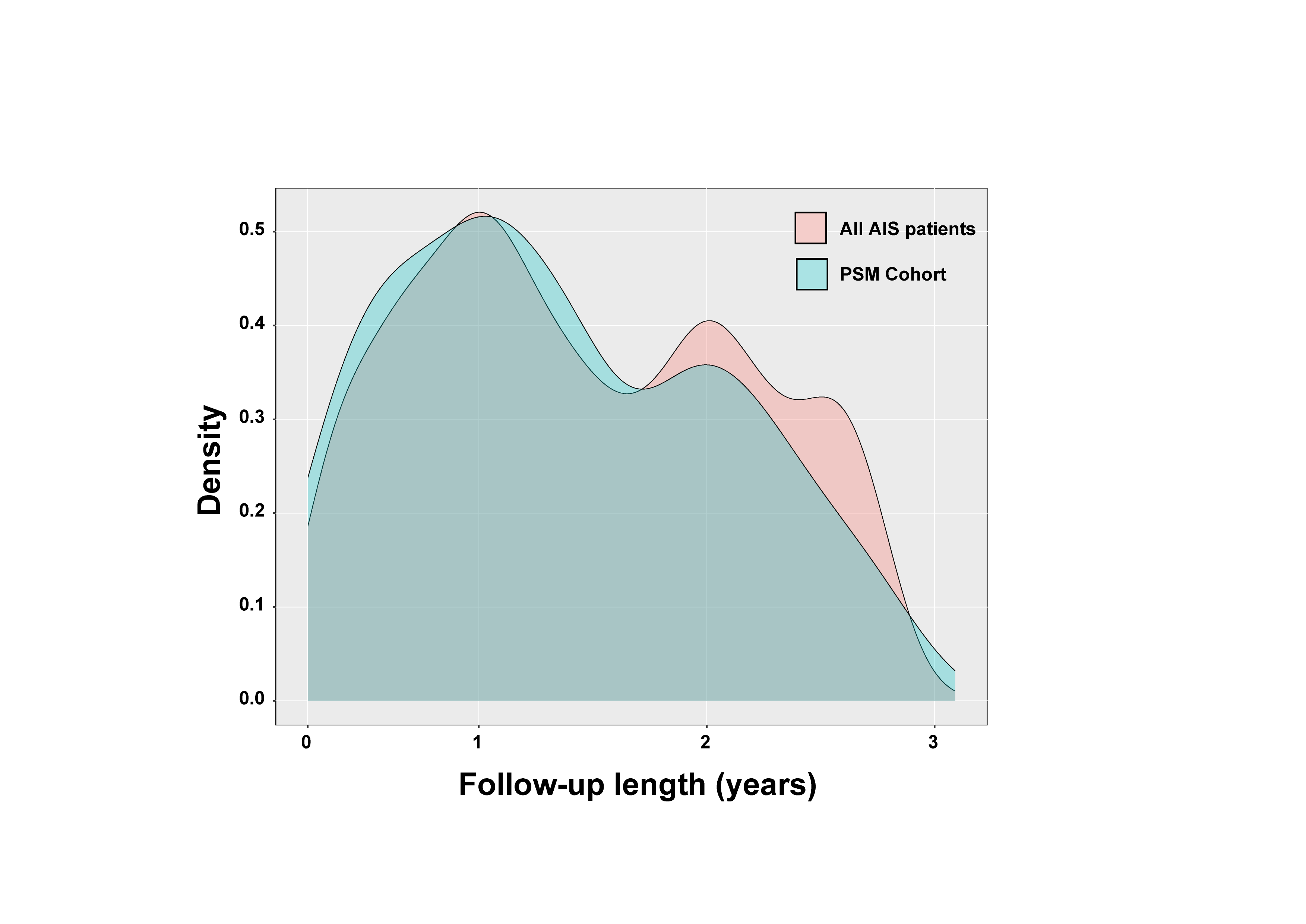

Supplement: SUPPLEMENTARY FIGURE S1 — Kernel density estimation-based distribution of follow-up times. [file Image_1.tif]
